# Controlled manual loading of body tissues: towards the next generation of pressure algometer

**DOI:** 10.1186/s12998-020-00340-7

**Published:** 2020-10-05

**Authors:** Davidk W. Evans, Alessandro Marco De Nunzio

**Affiliations:** 1 Centre of Precision Rehabilitation for Spinal Pain, School of Sport, Exercise and Rehabilitation Sciences, University of Birmingham, Birmingham, B15 2TT UK; 2 Research Centre, University College of Osteopathy, 275 Borough High Street, London, SE1 1JE UK; 3 LUNEX International University of Health, Exercise and Sports, 50, avenue du Parc des Sports, L-4671 Differdange, Luxembourg

**Keywords:** Clinical examination, Psychophysical, Force, Pressure, Algometer, Pain threshold, Temporal summation

## Abstract

Assessing the responses of body tissue subjected to mechanical load is a fundamental component of the clinical examination, psychophysical assessments and bioengineering research. The forces applied during such assessments are usually generated manually, via the hands of the tester, and aimed at discreet tissue sites. It is therefore desirable to objectively quantify and optimise the control of manually applied force. However, current laboratory-grade manual devices and commercial software packages, in particular pressure algometer systems, are generally inflexible and expensive. This paper introduces and discusses several principles that should be implemented as design goals within a flexible, generic software application, given currently available force measurement hardware. We also discuss pitfalls that clinicians and researchers might face when using current pressure algometer systems and provide examples of these. Finally, we present our implementation of a pressure algometer system that achieves these goals in an efficient and affordable way for researchers and clinicians. As part of this effort, we will be sharing our configurable software application via a software repository.

## Introduction

Assessing the responses of body tissue subjected to mechanical load is a fundamental component of the clinical examination, psychophysical testing and bioengineering research. The aim of loading may be to measure the mechanical properties of a body tissue: strength, resistance, elasticity or viscosity. Alternatively, the load can act as a stimulus, used to evoke responses from innervated receptors embedded within the tissue. Whatever the aim, accurate force application is essential if inferences are to be drawn from responses to tissue loading.

The forces applied during such assessments are usually generated manually, via the hands of the tester, and aimed at discreet tissue sites. Some testers have utilised computer-controlled, automated force application, via devices comprising pneumatic pumps [39] or force actuators [23, 45]. Whilst such automated devices will undoubtedly improve accuracy of force generation, cost and time constraints leave manual application as the most likely implementation for the foreseeable future. It is therefore desirable to objectively quantify and optimise the control of manually applied force. However, current laboratory-grade manual devices and commercial software packages are too expensive for most clinics (and many research departments). Furthermore, these devices do not always communicate directly with third-party computer hardware for data acquisition and analysis. Hence, the aim of this paper is to share our thoughts, experience and solutions in the development and implementation of a software application for both clinical and research environments, which is both flexible in its connectivity and generic in that it can be used with a variety of force measurement hardware.

## Principles

There are several principles that should be implemented as design goals within a flexible, generic software application for a controlled force application system, given currently available force measurement hardware. We introduce and discuss these below, providing examples that highlight the limitations of some current systems.

### Spatial targeting

During pressure testing in both clinical and research environments, load is typically applied to one site at any particular time. When pressure is applied to a body surface, force must first act upon the most superficial tissue at the site of application (e.g. epidermis of the skin when externally applied). Increasing force will sequentially and cumulatively act upon deeper tissues (e.g. dermis of the skin, in addition to subcutaneous fat, then also fascia, skeletal muscle, etc.), the precise nature of which will depend upon the composition of the underlying tissue [12, 13]. Commonly, the tester will want force to act upon tissue from a specific organ system (e.g. skeletal muscle, bone, or viscus), and must choose the application site at the body surface accordingly, usually identified via anatomical landmarks. It is usual practice to apply force perpendicular to the surface of the outermost tissue, the primary reason for this being negligible friction between the skin and the tissues beneath [6]. Force applied parallel to the surface is usually not met with significant resistance from underlying tissue, so unidimensional force measurement is sufficient for most pressure testing purposes. In addition, because there can often be several tissues simultaneously under the action of a perpendicularly applied force, a useful strategy might be to measure only the additional force applied once the target tissue (e.g. muscle) begins to provide resistance or can be seen to deform if using real-time imaging such as ultrasound (e.g. [14, 27]). Thus, the ability to calibrate or ‘zero’ the force recording, and automatically take note of the ‘baseline’ force at such a point, is deemed a useful feature for a software solution.

### Continuously ascending load

When applying force at a static, single site, it is difficult to avoid using an *ascending* approach, in which the magnitude of the load begins at zero and continuously increases over time. When establishing an important instant or threshold along the ascending load pathway, the rate of force increase will usually be linear. If testing tissue failure, which in human tissue is usually performed in cadaveric specimens, force should be able to ascend without interruption. However, when tissue failure is not the purpose of the test, particularly when the subject is intact, sentient and conscious, a useful feature is the option to set a safety limit to indicate the maximum pressure deemed both safe and ethical to deliver. The decision on where this limit ought to be set should be based on previous data relating to tissue failure limits and the current health and sensibility of the subject. If such data are unavailable, subject feedback, tester experience and common sense must be relied upon. Once the peak force is reached, it is usual to remove the loading effort either linearly or with immediate effect. Hence, the resulting force-time profile of this loading cycle will resemble a triangle, the area of which (half the product of total time duration and peak force) equates to the applied *impulse*, which is dimensionally equivalent to momentum and measured in newton-seconds (Ns).

### Quantitative sensory testing (QST) example: pressure pain threshold testing

QST involves the determination of discreet response thresholds or continuous stimulus-response profiles for sensory processing under normal and pathophysiological conditions [3]. A variety of quantifiable stimulus modalities (mechanical, thermal, chemical and electrical) can be applied to different tissues (e.g. skin [18], skeletal muscle [16, 41], teeth [42] and viscera [4, 11]) to evoke responses for the purposes of clinical examination or research data collection. One of the most common applications within QST is the provocation and quantification of pain from pressure stimuli; typically, an externally generated force is applied to a body site to stimulate receptors embedded within the underlying tissues to determine if and when pain is provoked. Indeed, departures from normative values are essential components of established diagnostic criteria [44]. Devices that apply and simultaneously measure pressure to evoke pain are called ‘pressure algometers,’ which are usually pistol-shaped with an operator handle and a single protruding probe that is applied to the subject’s body surface. Pressure stimuli are ordinarily applied at static, individual sites when using algometers. Nevertheless, other loading regimens are feasible: moving the site of pressure while loaded, using a wheel probe [15, 20] or a sliding probe [1, 26]; multiple site stimulation through gripping opposite sides of a digit [7], or an inflatable tourniquet cuff that applies circumferential pressure around an entire limb [27, 34].

**Fig. 1:**
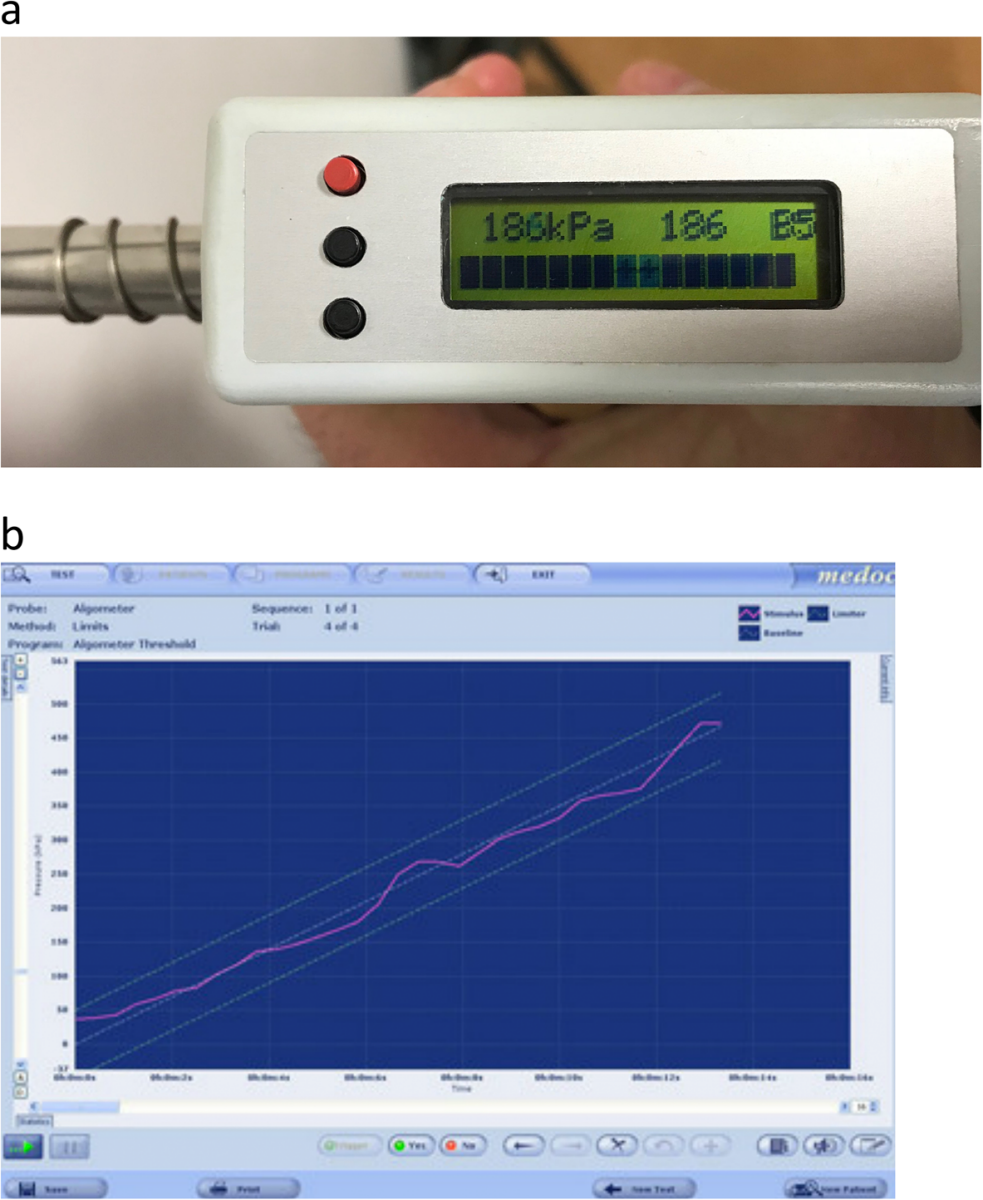
**a** Loading rate visual feedback provided by existing laboratory-grade pressure algometer systems (Somedic ‘Algometer 2’). **b** Loading rate visual feedback provided by existing laboratory-grade pressure algometer systems (Medoc ‘AlgoMed’)

Pain thresholds are a commonly used parameter within QST; the pressure pain threshold (PPT) is the minimal amount of pressure applied to one or more body sites that induces a painful sensation [17]. The most frequently employed method to measure pain thresholds involves increasing the stimulus at a constant rate until pain is evoked, known as the *ascending method of limits*. It is widely acknowledged that the *rate* of force development can affect the response to a pressure pain threshold test [25]; usually a higher loading rate will induce pain at a smaller force magnitude and produce a lower pain threshold result. The rate of force increase should therefore be controlled throughout the entire test. One way to control this loading rate when force is manually applied is through real-time feedback of the force being applied by the tester, with simultaneous guidance for the *pathway* of the force that is to be delivered through the entire test. When provided, such feedback and guidance is typically in visual format; via a small LED screen on the device such as the Somedic ‘Algometer 2’ (Somedic, Sweden) (Fig. 1a), or displayed on a connected computer monitor such as the Medoc ‘AlgoMed’ (Medoc, Israel) (Fig. 1b).

All commercially available algometers will automatically capture the greatest magnitude (peak) of force achieved during a loading cycle. Laboratory-grade algometer systems can additionally accommodate a handheld ‘trigger’ button, so that subjects can instantiate the magnitude of force associated with the precise moment of their threshold response. This is useful since there will always be a time-lag between a subject first perceiving the stimulus, communicating their response to the tester, and the tester reacting to this signal. Whilst most laboratory-grade algometers can accommodate a trigger button, these are generally not configured to record multiple response instants within the same ascending load cycle. However, a tester might want to record the force and associated time when the sensation of touch or pressure is first experienced by the subject (detection threshold), in addition to when the pressure becomes painful (pressure pain threshold), and also when the increasing pain becomes intolerable (pain tolerance). The option to use the trigger button to record multiple instants within a single ascending load cycle is therefore a useful feature.

Discreet force-time instants are not the only responses that a tester might seek to measure during a loading cycle. With the exception of the Medoc ‘Algomed’ device, current laboratory-grade algometer systems do not typically provide an option to simultaneously record a subject’s continuous stimulus-response throughout the entire test (e.g. real-time pain intensity via an electronic visual analogue scale). An implementation of this feature requires the ability to capture multiple data sources and to do so at identical sampling frequencies. Furthermore, it is conceivable that testers will want an immediate summary of their primary test results, especially if multiple test repetitions are to be performed at the same site and results of previous tests (e.g. mean peak force) are to be used to inform parameters of the next. However, most algometer systems are not configured to automatically summarise multiple tests, thus forcing the tester to physically remove the algometer probe from the testing site, make a note of their latest results, perform some calculations, reset the equipment, and generally interrupt the assessment process. When an assessment should consist of multiple uninterrupted test repetitions, an option to display an automatic summary of previous test results would certainly be a useful feature.

### Ascending/descending load cycles

**Fig. 2:**
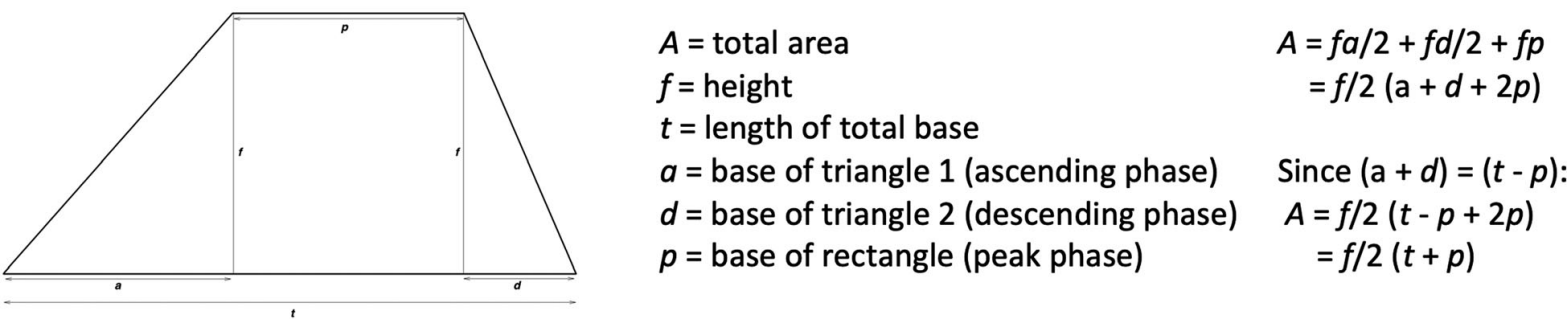
The trapezoid shape of each impulse within a three-phase loading cycle

Unlike uninterrupted ascending loading, a very different pressure testing regimen is one or more deliberately interrupted (limited) loading cycles. Each cycle will consist of a minimum of two discreet phases: an ascent and a descent. An ascending load is first applied until a pre-designated peak force is attained (‘ascending’ phase). After this, the peak force can be maintained for a given duration (‘peak’ phase); this duration can be zero, making the phase instantaneous. Finally, the force is then returned to a baseline value (‘descending’ phase), which does not have to be zero if there are multiple loading cycles. When all three phases are present, the force-time profile of each complete loading cycle will take the form of a trapezoid; the area of each trapezoidal cycle equates to the impulse applied, which can be calculated using the equation derived in Fig. 2. Basic geometry shows that a trapezoid can be constructed from two right-angled triangles and a rectangle, all of equal height (*f*), and the area calculated as such. If the duration of the peak phase (*p*) is zero, the trapezoid profile will reduce to that of a single triangle and the area calculated accordingly.

These trapezoidal (or triangular) loading cycles can be repeated multiple times within a single series, according to the aims and objectives of the test. Such a repetitive loading regimen could be used to test the failure limits of a tissue by fatiguing it with multiple load cycles; this is unlikely to be tested in vivo with human subjects. Alternatively, the cumulative ‘dose-response’ effect of static or repetitive sensory stimuli can be evaluated as part of a QST assessment.

### QST example: pressure temporal summation

A repeating series or ‘train’ of suprathreshold stimuli upon innervated tissue in conscious, sentient subjects typically results in a non-associative learning response: either a progressive response increment (sensitization), or a progressive response decrement (habituation). When the stimulus is above the threshold required to activate nociceptors and the interval between impulses is short (within a few seconds), sensitization will usually occur. This particular type of sensitization is called temporal summation [2].

**Fig. 3:**
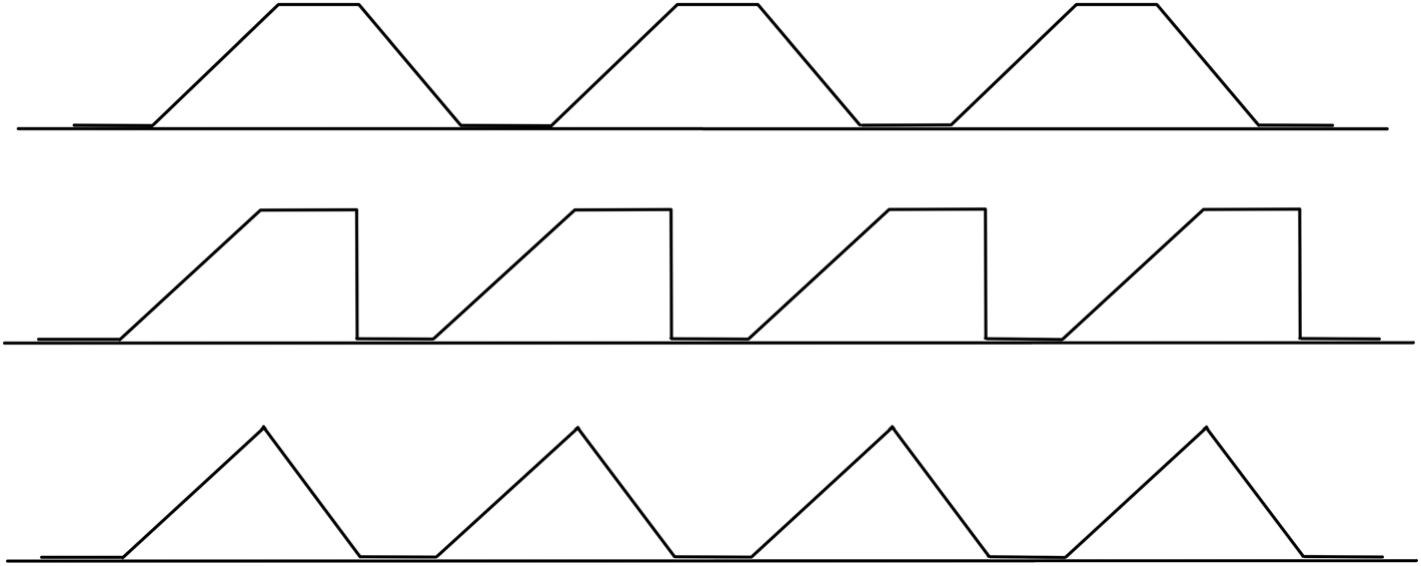
Example force-time diagrams of repetitive temporal summation stimuli

The neurophysiological phenomenon responsible for temporal summation occurs within the spinal cord and is known as ‘wind-up’. It is primarily due to the relatively long duration of excitatory synaptic potentials evoked from stimulated C-fibre nociceptors [24, 35]. If a stimulus is repeated before the voltage at the postsynaptic membrane has fully recovered from the preceding stimulus, then a cumulative increase in postsynaptic voltage will result, which raises the likelihood of an action potential. Temporal summation can be utilised as a QST assessment of sensory excitability in the spinal cord, during which resulting nerve impulses and/or subjective responses are measured. There are several parameters within a series of stimuli that can be varied to produce temporal summation (Fig. 3): the *amplitude* of stimulus; *time duration* of stimulus; time duration between stimuli (known as the *inter-stimulus interval*); and, *number of stimuli*. When using mechanical stimuli to evoke temporal summation, the stimulus amplitude will be the magnitude of applied pressure, which can be reduced to the magnitude of applied force when the area of application is kept constant.

In a typical example of pressure temporal summation, ten force-time impulses might be delivered upon the skin overlying a skeletal muscle, and the response to be measured throughout the test the real-time perceived intensity of pain (e.g. via a visual analogue scale of 0–100, where 0 = ‘no pain’ and 100 = ‘worst pain imaginable’). Individual impulses within any single series of stimuli are usually identical; hence, an approach that undoubtedly provides comparable data between individuals is to deliver impulses with exactly the same trapezoidal profile to every participant. However, pressure pain thresholds at equivalent body sites are known to vary widely between individuals [9, 28, 29, 31]; consequently, a tester might instead decide to *individualise* the trapezoid to every person, and even every site, ensuring that stimuli are always suprathreshold. To do so, the tester would first have to measure the pressure pain threshold for that particular site (as described above), and then use this result to set the target peak force for all subsequent impulses at that site (e.g. [37]). Typical values for remaining parameters would be to hold the maximum stimulus amplitude (i.e. the peak force) for a duration of 1-s, then immediately reduce the stimulus amplitude to zero (i.e. the descending phase will possess negligible force-time area), and finally pause for a 2-s inter-stimulus interval period before commencing the next identical impulse, continuing this process until all ten impulses have been delivered.

Two interpretations (we can call these Methods 1 and 2) can be made when individualising temporal summation stimuli to implement the above example. Let us imagine a study in which an equivalent body site was tested in three hypothetical participants (A, B and C). In both Methods, the peak force will be individualised to each participant using their pressure pain threshold value measured at that particular site: the peak force used for Participant B will be 10% greater than Participant A, and that of Participant C will be 10% greater than Participant B (and thereby 21% greater than Participant A). In Method 1, all participants will be tested using impulses of the *same time duration* (5-s ascending, 1-s maintaining the peak force, 0-s descending). By contrast, in Method 2 the ascending phase will be individualised by employing the same approach utilised during pain threshold testing: a *constant loading rate* (of 20 N/s).

In Method 1, by keeping both the peak force and time duration of each loading cycle constant and independent of one another, the loading rate during the ascending phase would be allowed to vary between subjects as a function of just the peak force. Since ascending and descending phases each take the form of a triangle on the force-time diagram (Fig. 2), their respective areas are equal to half of the product of their peak force and time duration. In both Methods, the peak phase is a simple rectangle on the force-time diagram; a product of the magnitude of peak force and the time duration of this phase. Hence, in Method 1 the total magnitude of impulse (the ‘area under the curve’) delivered during each loading cycle will remain proportional to the peak force throughout all phases (Table 1). For Participant A, with their individualised peak force set at 100 N and negligible impulse delivered during the descending phase, the total impulse delivered through one complete loading cycle (sum of ascending and peak phases) is 350 Ns. For Participant B, with their peak force set at 110 N, the total impulse applied will be 375 Ns (an increase of 7% compared to Participant A), and for Participant C, with their peak force set at 121 N, the total impulse applied will be 402.5 Ns (an increase of 15% above Participant A, and 7% above Participant B). Additionally, since the time duration of all phases are constant, the total duration of all ten identical impulses separated by nine 2-s inter-stimulus intervals will be 240.0-s for all participants.

**Table 1:** Impulse delivered during a loading cycle with Method 1: using a constant time duration for every phase

Phase	Ascending phase	Peak phase	Descending phase	Totals
Peak force	= f (constant)	= f (constant)	= f (constant)	= f
Time	= a (constant)	= p (constant)	= d (constant)	= t (constant) = a + p + d
Loading rate	= f/a (variable)	= zero (constant)	= f/d (constant)	
Impulse	= f*a/2	= f*p	= f*d/2	= f*a/2 + f*p + f*d/2 = f(a + 2p + d)/2 **= f(t + p)/2**

Method 2 maintains a constant loading *rate* during each ascending phase. The loading rate is not only proportional to the peak force, but also to the time duration of the ascending phase, which is itself a function of the peak force. The impulse delivered during the ascending and descending phases of Method 2 will therefore be directly proportional to the square of the peak force (Table 2). As a result, in our example the total impulse delivered to Participant B (412.5 Ns) will be nearly 18% greater than the 350 Ns delivered to Participant A; by comparison, Participant C (487.0 Ns) will have received over 39% greater impulse than Participant A and 18% more than Participant B. Additionally, the time duration for the ten impulses with Method 2 would be 240.0-s for Participant A, 245.0-s for Participant B, and 250.5-s for Participant C. Furthermore, if the unloading rate during the descending phase was set as a mirror-image of the ascending phase, which is perfectly plausible in such a study, these hypothetical differences between Methods 1 and 2 would have doubled.

**Table 2:** Impulse delivered during a loading cycle with Method 2: using a constant loading rate during ascending and descending phases

Phase	Ascending phase	Peak phase	Descending phase	Totals
Peak force	= f (constant)	= f (constant)	= f (constant)	= f
Time	= a (variable) = f/r_a_	= p (constant)	= d (variable) = f/r_d_	= f*r_a_ + p + f*r_d_ (variable)
Loading rate	= r_a_ (constant)	= zero (constant)	= r_d_ (constant)	
Impulse	= f*a/2 = f(f/r_a_)/2 **= f**^**2**^**/2r**_**a**_	= f*p	= f*d/2 = f(f/r_d_)/2 **= f**^**2**^**/2rd**	**= f**^**2**^**/2r**_**a**_ + f*p + f^**2**^**/2rd**

Method 2 should *not* be used to individualise pressure temporal summation assessments. Firstly, since every subject will be given a different peak force, allowing a second parameter – the time duration of each impulse and consequently the entire test – to also vary between subjects will result in an incomparable and biased test. Secondly, although some readers might suppose that one can adjust the data by taking the square root of the results, especially if peak and descending phases provide negligible impulse, it cannot be assumed that neurophysiological responses to increased impulse magnitudes and longer durations will be linear [38]; such biological responses are often cumulative and likely logarithmic, so data transformations might not be sufficient. Importantly, laboratory-grade algometer systems currently provide only an option to set loading rates for the ascending phase, and typically these must be chosen from a limited selection of fixed rates; hence, peak force cannot be separated from the duration of the ascending phase and operators are forced to implement Method 2. Additionally, current algometers provide no option to configure either peak or descending phases, and no option for guidance and recording of multiple loading cycles. Hence, these legacy systems cannot be used to accurately apply individualised temporal summation stimuli; the ability to independently configure these settings is essential in any software implementation that is intended to do so.

## Implementation of an improved algometer system

**Fig. 4:**
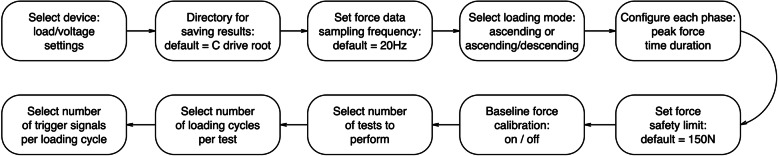
Configurations implemented in our software application

Given the above principles, the primary hardware and software components alongside corresponding features of our implementation of an improved, highly flexible algometer system are described below. Our configurable software application (Fig. 4) was developed using the LabVIEW software development platform (National Instruments, USA), which produced an executable file for our desktop computer running Windows 10 operating system (Microsoft, Seattle, USA).

### Hardware: force transduction and quantification

The applied force will need to be transduced into an electrical signal so that it can be accurately recorded. Transduction is usually performed by one or more load cell, which converts force into voltage. Many devices contain load cells, but those specifically designed to record applied force very accurately are digital *force gauges*. At the time of writing, there were several manufacturers of laboratory-grade digital force gauges and the cost of these generally compared favourably to the equivalent cost of laboratory-grade algometer hardware, presumably because generic force gauges are used across a wider variety of industries and therefore have a larger market. For our implementation, we chose a Mark-10 Series 7 (M7–100) digital force gauge (Mark-10, USA), capable of measuring both compression and tension forces, with a force range of +/− 100 lbF (= 200 lbF or 889.64 N total) and corresponding analogue voltage range of +/− 1.0 V (= 2 V total).

### Hardware: data acquisition and transmission

Like many laboratory-grade digital force gauges, ours has a variety of built-in options to connect with and transfer data to a computer, including digital (e.g. using USB ports) and analogue interfaces. According to the principles described previously, it is desirable to simultaneously aggregate multiple data inputs (e.g. measured force and participant responses). One option to do so is to pass analogue signals from each device through a data acquisition device, which aggregates and encodes data before transmission to a computer for processing and storage. Including a separate data acquisition device obviously increases the technical requirements and hardware cost but we believe the ability to aggregate multiple inputs is well worth the investment. In our implementation, we used a 16-bit data acquisition board (NI USB-6002, National Instruments, USA), which transmitted aggregated analogue data to the desktop computer via a USB connection. To improve portability, we chose a data acquisition board that derives power from its USB connection with the desktop computer (i.e. it does not need an external power supply to run).

### Software: configuration of load cell voltage conversion

The load cell within the force gauge transduces applied load into a specific voltage within a designated range. Within this range, the analogue load-voltage transduction relationship is usually linear, so the conversion factor can be calculated by dividing the range of voltage produced by the load cell (e.g. +/− 1.0 V = 2 V total) by the range of force measured by the device (e.g. +/− 500 *N* = 1000 N total); these examples produce a conversion factor of 0.002 V/N. The relevant information for any given device is usually found in manufacturer specifications; for our Mark-10 Series 7 (M7–100) force gauge, the conversion factor was 0.00225 V/N. It is important that any generic software solution is able to incorporate such device properties for correct interpretation and processing of analogue signals.

### Software: configuration of sampling frequency

We decided that force data sampling frequency should be adjustable within the software as requirements would vary with different loading tasks. For participant responses to pressure stimuli, we considered the time required for afferent inputs to be transduced and encoded within peripheral receptors, conducted along axons and transmitted to the central nervous system, processed and interpreted in the brain, before the participant can begin to signal their response. As such, a stimulus-response period of 150 ms or below (i.e. a frequency of 6.7 Hz or above) is widely considered insufficient for supraspinal processing of nociceptive reflexes ([43, 30]). Additionally, since the Nyquist-Shannon sampling theorem [32, 40] states that the sampling rate must be set at more than twice the highest analogue frequency of a signal (i.e. 13.33 Hz for this example), a default sampling frequency of 20 Hz was considered adequate for pressure pain testing.

### Hardware: force gauge accessories

**Fig. 5:**
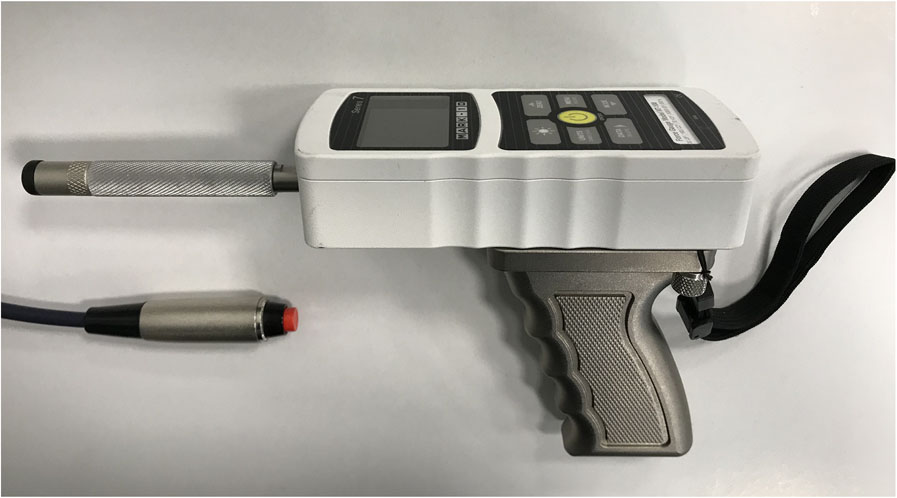
Force gauge with accessories

Most manufacturers of force gauges produce various optional specimen contact accessories that can be attached via screw thread to the probe. The manufacturer of our chosen force gauge (Mark-10, USA) produced a hard rubber tip with a surface area of 1.2 cm^2^ (accessory G1011, see Fig. 5). This tip was deemed particularly suitable for contact with body surfaces since it was manufactured with slightly rounded edges [19], which are known to deliver a more even force at the site of contact and facilitate loading of deeper tissues [12]. The rubber material was also relatively non-slip, which is advantageous during loading.

Initial testing showed that the casing of the force gauge would often come into contact with the participants skin during loading, because the distance from this casing to the end of the attached contact tip was very short. We therefore fabricated a knurled, cylindrical aluminium spacer to increase this distance (Fig. 5). Utilising custom-made spacers confers the additional advantage of allowing any contact accessory (potentially from any manufacturer) to be attached to the probe (e.g. tips of varying materials, shapes and sizes, Von Frey filaments, moveable wheel or ball, etc.). We also looked at various options for the operator handle and chose to equip the gauge with a pistol grip handle supplied by the gauge manufacturer (accessory E1010). Finally, we attached a generic wrist strap to reduce the likelihood of the operator dropping the gauge and damaging the onboard load cell.

### Hardware: participant responses

We incorporated a handheld, normally open, push button switch (Philmore, LKG Industries, USA) into our system (Fig. 5) so that participants could trigger an instantaneous signal to be automatically recorded; we configured the software so that one or more trigger events could be recorded during a task. We also configured the software to allow the participant to use the same button to remotely initiate the commencement of a task, in the event that the operator has the force gauge positioned ready for testing and is then unable to simultaneously operate the computer mouse or keyboard. To facilitate testing beyond instantaneous trigger signals, we fabricated an electronic visual analogue scale (eVAS) using a 100 mm linear potentiometer, which would enable us to incorporate continuous participant stimulus-response data. All participant response instruments were connected directly to the data acquisition device.

### Software: configuration of ascending load mode

The rate of force increase (ascending loading rate) can be set in *ascending load mode*. Although the hardware was not limited in any way, a safety limit guideline was added to the software; the default value for this limit was set at 150 N, which converts to 1000 kPa when used with the 1.2 cm^2^ contact tip. This value is comparable with the limits of other algometers and well below estimates for skin failure limits [10, 21, 22, 33, 36].

Since pain thresholds are often measured using the average of multiple measurements at a single site [5, 8], we included the ability to pre-set the number of test repetitions and the time between these repetitions (the inter-test interval). The software was configured to automatically calculate and then display the mean of the peak force applied during a preconfigured series of tests, as well as the mean of the first trigger response from a series. We also included an option to calibrate and ‘zero’ the applied force at the commencement of a task to take account of any preparatory loading and/or the combined weight of the aluminium spacer and contact tip. This baseline value is automatically recorded.

### Software: operator feedback and guidance

**Fig. 6:**
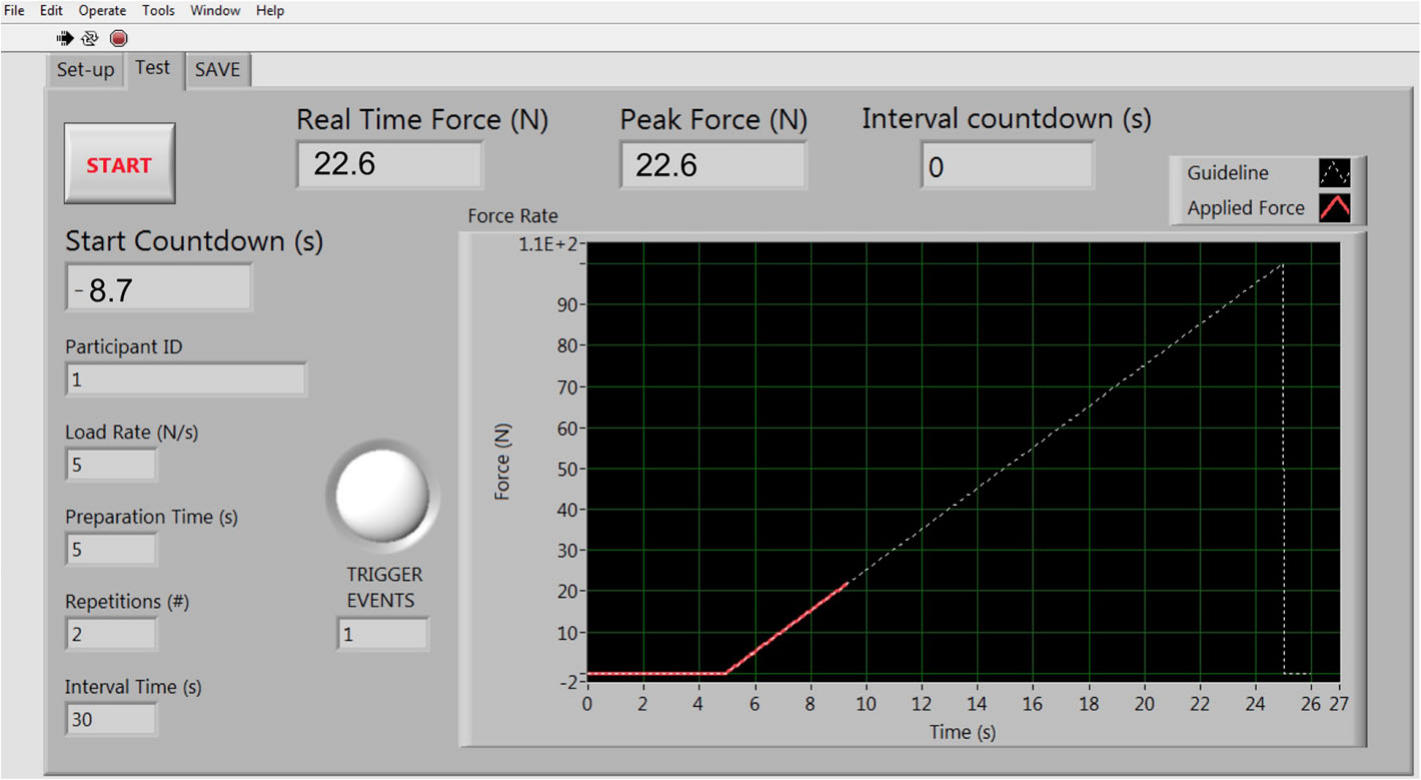
Visual real-time feedback and guidance during ascending load mode

Consistent with current algometer systems, we ensured that our system provided real-time visual feedback of the applied force superimposed over a guideline for the intended force pathway, all displayed on a computer monitor (Fig. 6). The predesignated safety limit appears as a visible horizontal red line on the monitor, giving advanced notice to the tester to not apply force beyond this magnitude. Since this visual feedback and guidance was intended to dominate the attention of testers, we added a flashing red ‘light’ to the software display, as well as a simultaneous audible ‘beep’ routed through the sound card of the computer, to signal the initiation of a trigger response from the subject and prompt appropriate actions from the tester.

### Software: configuration of ascending/descending load mode

Besides all of the configurable settings described above, additional settings were required for *ascending/descending load* mode. Unlike the uninterrupted *ascending load mode*, this mode consists of one or more loading cycle per test. Hence, a setting for the number of loading cycles per test was added. In addition, if multiple loading cycles are selected, the time duration between cycles (inter-stimulus interval) can be set. Settings to establish individual phases of the trapezoidal force-time guideline were also added: the target peak force (limit for ascending phase), the duration that the peak force is to be maintained (peak phase), and the rate of force unloading (descending phase) could all be configured independently.

### Software: outputs

The software was configured so that all test results are automatically saved, within a datafile in CSV format, to a chosen directory on the computer (default being the C-drive root directory). All preconfigured settings are saved at the head of the datafile, as are the date and time of testing, participant number, and any operator notes required at the point of saving the test data (e.g. participant asked to stop testing). All input values (i.e. force, eVAS and any trigger responses) are continuously recorded and timestamped throughout the sampling timeframe. Summary results, including peak values, mean values, and area under the curve per loading cycle, are automatically calculated and recorded for all of these continuous data inputs. Force and eVAS values are also automatically summarised for any trigger instants. The baseline value is recorded if force is zeroed at task commencement.

### Requirements

The above setup requires a considerable investment in hardware (approximately US$3000 in 2018) and some technical knowledge and skills, particularly in relation to the data acquisition device (although manufacturers do provide instructional resources). Development of the software application involved significant planning and technical knowledge. We have therefore decided to share this application via a software repository, where we intend to provide user instructions, updates and enhancements over time, and welcome user feedback and suggestions: https://github.com/usetheforcegauge/forceguider.

## Conclusions

Basic principles that should be considered when using mechanical loading during clinical examination and laboratory assessments have been introduced and discussed. We have looked at specific clinical examples where current pressure algometry systems are used and where they are not suitable. We have also highlighted solutions to these limitations and implemented these into a flexible pressure algometer system, which we have described in detail.

We believe that the principles, design goals and implementation of a more flexible pressure algometer system described in this paper represent an improvement upon existing commercial systems. We hope that this will encourage manufacturers to develop the next generation of pressure algometer systems and encourage researchers and clinicians to consider using more affordable and configurable generic force measurement equipment, such as digital force gauges.

## Data Availability

This is not applicable since we are not reporting research data.
